# Multidisciplinary management of a rare case of a large functional paraganglioma in the posterior mediastinum in an adolescent: a case report and literature review

**DOI:** 10.3389/fonc.2025.1651878

**Published:** 2025-10-03

**Authors:** Li Hang Liao, Xiao Jun Du, Jian Ma, Zhang Yong Ren

**Affiliations:** Department of Thoracic Surgery, The Affiliated Hospital of Guizhou Medical University, Guiyang, Guizhou, China

**Keywords:** pheochromocytoma, mediastinal tumor, paraganglioma, adolescent, surgical resection, multidisciplinary collaboration

## Abstract

A pheochromocytoma (PPGL) in the mediastinum can originate from the paravertebral sympathetic nerve chain in the posterior mediastinum, or from the intervertebral, subclavian, coronary, pulmonary, or paravertebral paraganglia in the anterior or middle mediastinum. This case report presents the diagnosis of a large, functional paraganglioma in the posterior mediastinum, accompanied by excessive catecholamine secretion, in a 14-year-old male patient. Through multidisciplinary collaboration, radical tumor resection was performed with successful outcomes. This study systematically summarizes the standardized diagnostic pathway and key surgical techniques for this rare disease. The patient exhibited symptoms of exercise-induced retrosternal pressure and paroxysmal palpitations. A chest CT scan prior to surgery revealed a large tumor in the posterior mediastinum, measuring 8.0 × 5.6 × 7.2 cm, which was wrapping around the descending aorta. During the surgical procedure, it was demonstrated that an acute hypertensive crisis was precipitated by the presence of tumor traction and compression. Despite rigorous pharmacological intervention, blood pressure levels were highly variable (ranging from 235/172 mmHg to 36/26 mmHg). Subsequent pathological analysis after surgery confirmed the diagnosis of a functional paraganglioma, as indicated by strong positive CgA expression as determined by immunohistochemical staining. This case study provides a detailed account of managing a posterior mediastinal paraganglioma, emphasizing the dual challenges of diagnosis and treatment. The first challenge is definitively determining the patient’s functional status through multimodal imaging combined with biomarkers. The second is optimizing an α-receptor blockade regimen and developing dynamic blood flow management strategies intraoperatively to prevent life-threatening complications.

## 1 Introduction

It is estimated that 10% to 20% of patients diagnosed with PPGL will develop metastatic disease (1). In view of its high malignant potential, patients with localized disease should undergo radical surgical resection. However, it is important to note that surgery carries a significant risk and requires meticulous perioperative management by a specialized multidisciplinary team at a center with extensive expertise (2, 3). The enhancement of clinical management, surgical techniques and anesthetic support has been demonstrated to reduce the mortality rate of functional PPGL surgery to between 0% and 2.9% (4, 5). Paragangliomas have been reported principally in adult patients in the retroperitoneum (6), bladder (7), head (8), and heart (9). However, cases of paragangliomas in the posterior mediastinum of the thoracic cavity in adolescents are extremely rare. It is important to note that adolescent patients often have elevated catecholamine levels due to their increased physical activity. However, this heightened activity can result in associated symptoms being overlooked inadvertently. Due to their deep anatomical location and rich blood supply, posterior mediastinal lesions are highly prone to catastrophic hemodynamic instability during surgery. This case report presents a systematic analysis of the atypical clinical manifestations, multimodal imaging assessment, radical surgical strategy and optimized perioperative management protocol of a giant functional paraganglioma in a 14-year-old male patient, highlighting the typical challenges in the diagnosis and treatment of such tumors.

## 2 Case report

A 14-year-old male presented with a 2-year history of a posterior mediastinal mass, which demonstrated progressive enlargement (5 cm to 8 cm on serial imaging) and new-onset retrosternal oppression with paroxysmal palpitations during exertion. It is noteworthy that no cases of hypertension, headaches, or visual disturbances were observed. Family history: No history of pheochromocytoma/paraganglioma, related endocrine tumors, early-onset hypertension, or sudden death was identified over three generations. Preoperative laboratory investigations: Complete blood count, electrolytes, liver/renal function, coagulation profile, fasting blood glucose, glycated hemoglobin, and thyroid function showed no abnormalities. Specific biochemical tests for PPGL were not performed.

### 2.1 Contrast-enhanced thoracic CT

**Figure 1:**
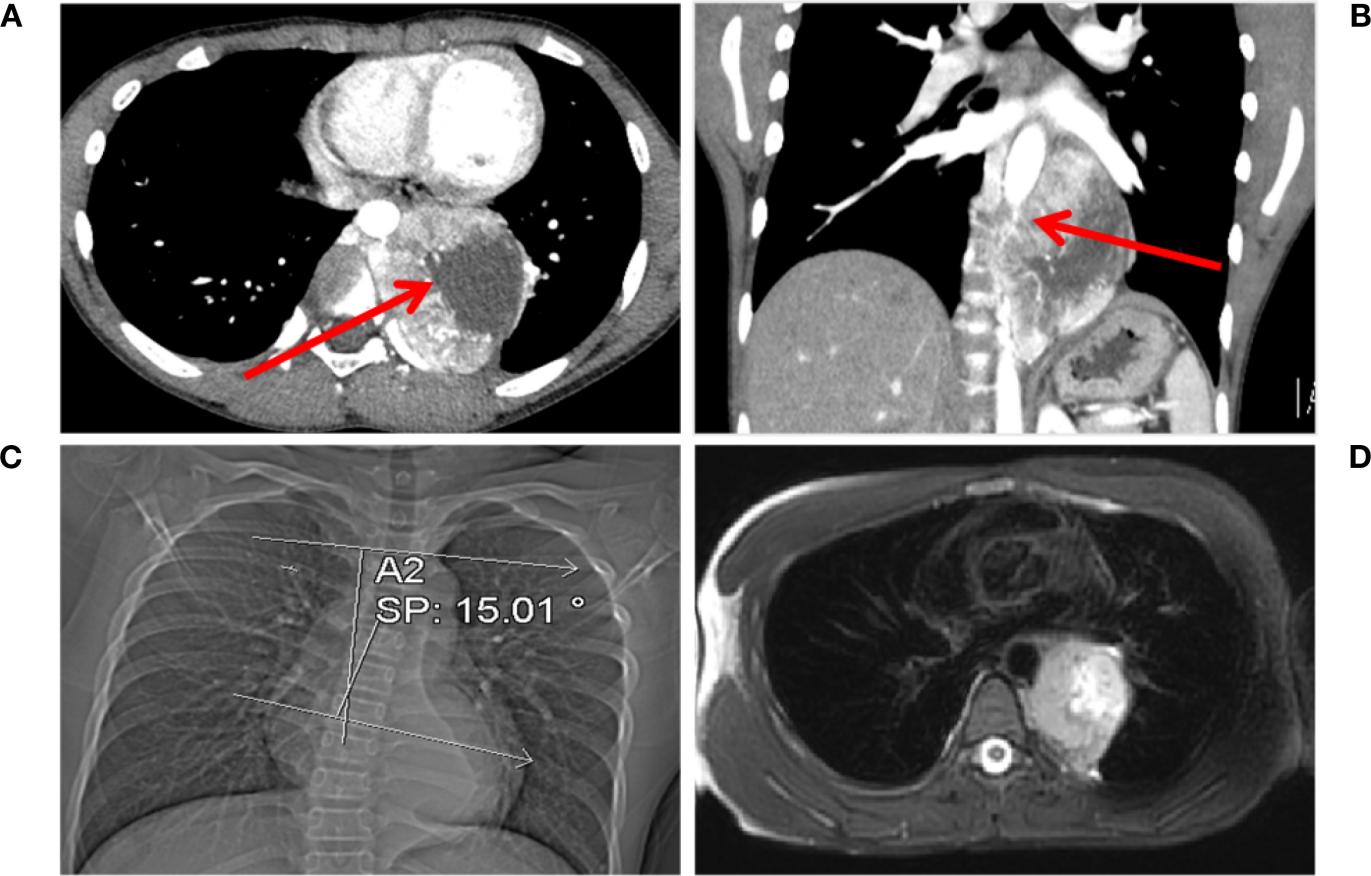
Chest CT and MRI findings. **(A)** Multiple vascular shadows are visible in the arterial phase, accompanied by an irregularly enhanced mass. Necrosis is present within the tumor, as indicated by the red arrow. **(B)** The tumor encircles the aorta (indicated by the red arrow) in the coronal view. **(C)** The tumor compresses the spine, causing scoliosis (Cobb angle: 15.01°). **(D)** T2-hyperintense lesion with clear margins is observed, with no involvement of the intervertebral foramen or spinal cord.

The mass, measuring 8.0 × 5.6 × 7.2 cm, exhibits cystic necrosis and is heterogeneously enhanced (**Figure 1A**). The arterial feeders originate from the descending aorta. The mass demonstrates circumferential aortic encasement, accompanied by spinal column displacement (**Figure 1B**). This results in the induction of a Cobb angle scoliosis measuring 15.01° (**Figure 1C**). The MRI scan revealed a lesion that was T1-hypointense and T2-hyperintense, with clearly defined margins. Importantly, there was no evidence of involvement with the foramina or spinal canal (**Figure 1D**). Enhanced chest CT and MRI scans clearly showed the extent of the lesion and its vascular relationships. No other suspicious lesions were identified in the chest or abdomen.

### 2.2 Initial diagnosis

**Figure 2:**
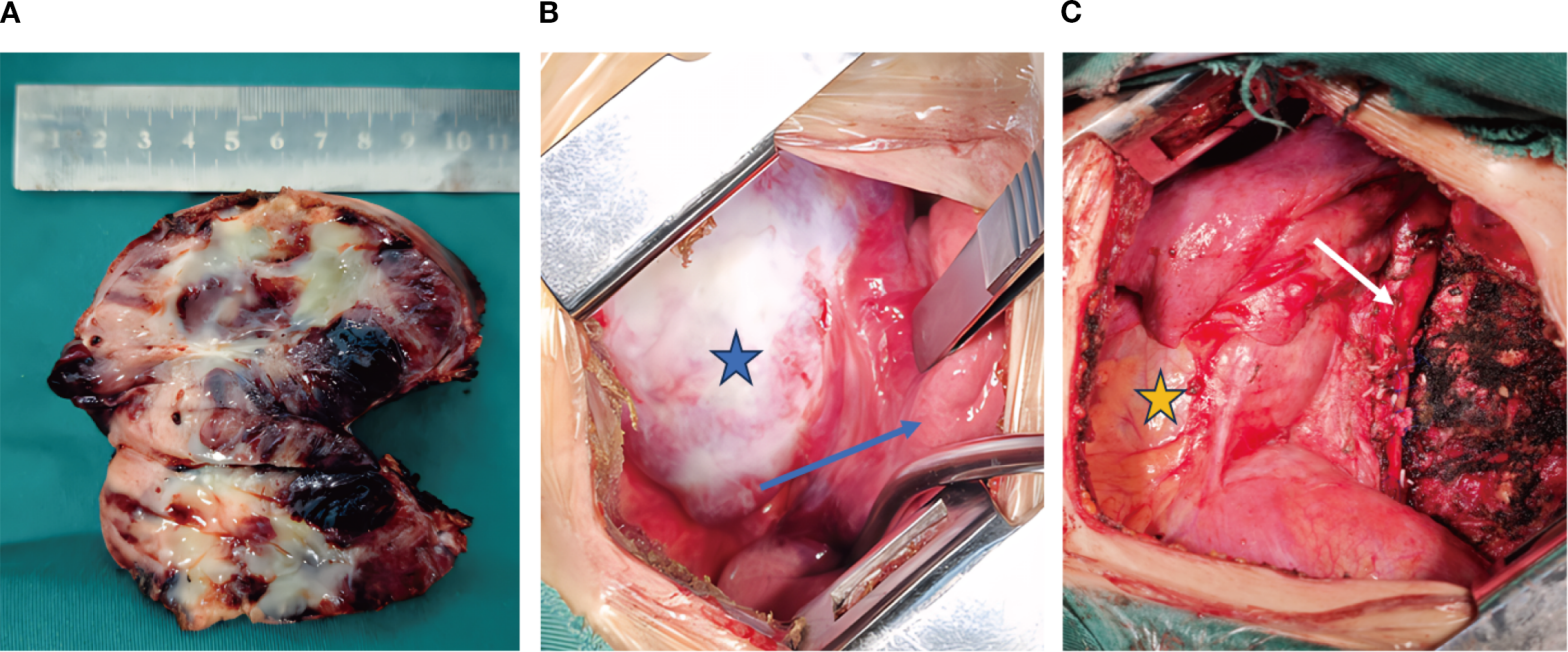
Intraoperative findings. **(A)** The tumor is approximately 10 cm long and has a complete capsule. Focal areas of necrosis can be seen within the tumor. **(B)** The tumor capsule is intact (indicated by the blue pentagram), and the tumor is tightly adherent to the adjacent lung parenchyma (indicated by the blue arrow pointing to the lung). **(C)** The tumor after resection in the thoracic cavity (the white arrow indicates the aorta, and the yellow pentagram indicates the pericardium).

**Figure 3:**
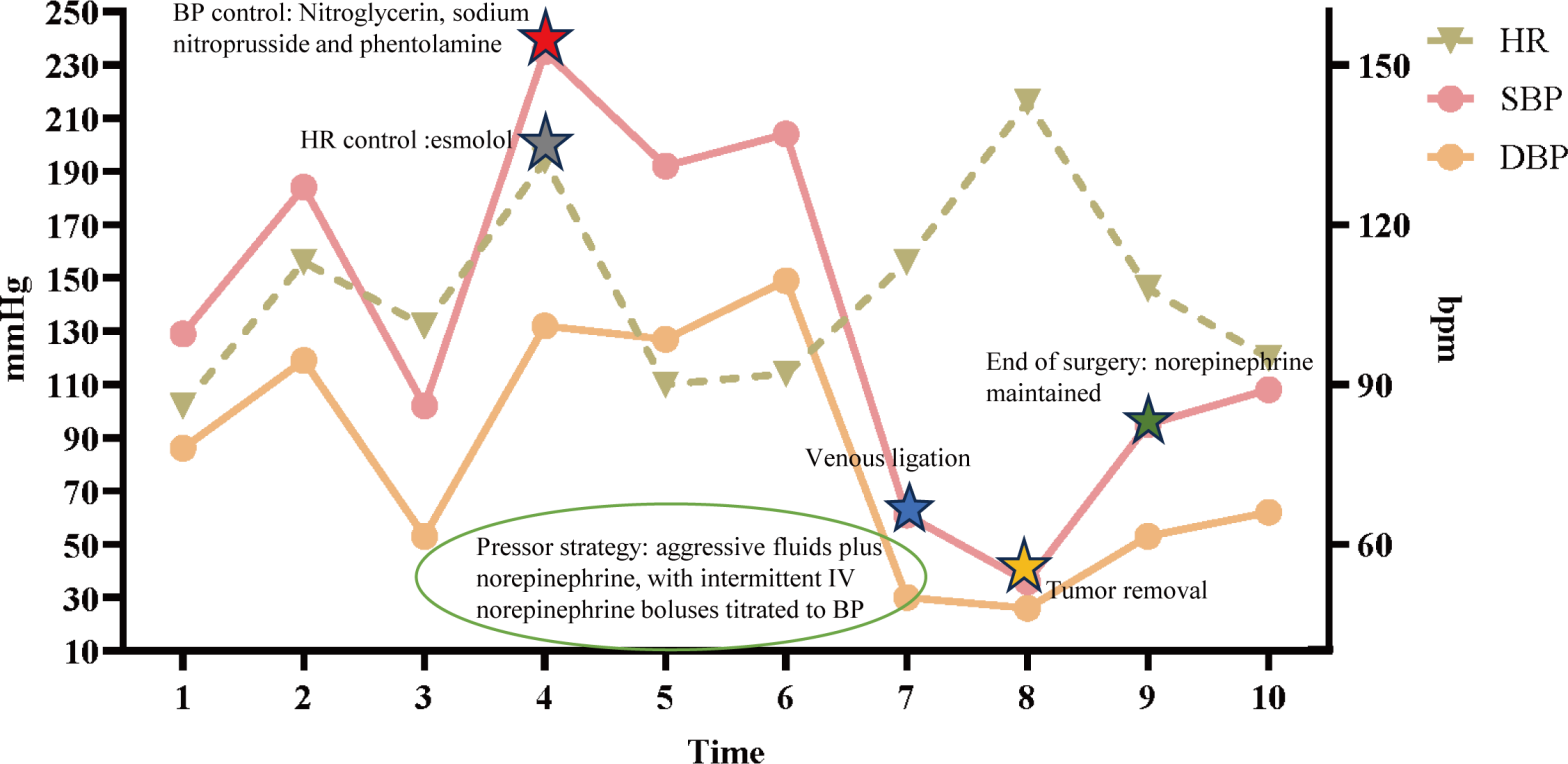
Intraoperative hemodynamic timeline. Left y-axis: arterial pressure (mmHg); right y-axis: heart rate (bpm). Curves depict systolic blood pressure (SBP), diastolic blood pressure (DBP), and heart rate (HR) over time. The x-axis marks key intraoperative time points (1–10): 1 operating-room entry; 2 induction of anesthesia (anticipated BP/HR decreases; low-dose norepinephrine 0.02 mg/mL infused at 5 mL/h to maintain perfusion); 3 incision/start of surgery (norepinephrine continued as above); 4–5 tumor traction and arterial ligation [BP control: nitroglycerin 0.3 mg/mL at 2 mL/h, sodium nitroprusside 0.01 mg/mL at 2 mL/h, phentolamine IV bolus 0.03 mg; rate control: esmolol 1 mg/mL at 5 mL/h]; 6 aortic rupture with massive hemorrhage (concurrent BP/HR control with emergent transfusion and large-volume fluid resuscitation to maintain circulating volume); 7–8 venous ligation and tumor removal with abrupt hypotension (pressor strategy: aggressive fluids plus norepinephrine 0.04 mg/mL at 35 mL/h, with intermittent IV norepinephrine boluses titrated to BP); 9 end of surgery (norepinephrine 0.04 mg/mL maintained at 35 mL/h); 10–48 h postoperatively, normotension without vasoactive support. Given marked BP lability, the anesthesia information system could not provide minute-to-minute pump-rate granularity; representative rates and time windows are displayed. HR, heart rate; SBP/DBP, systolic/diastolic blood pressure; bpm, beats per minute; mmHg, millimeters of mercury.

**Figure 4:**
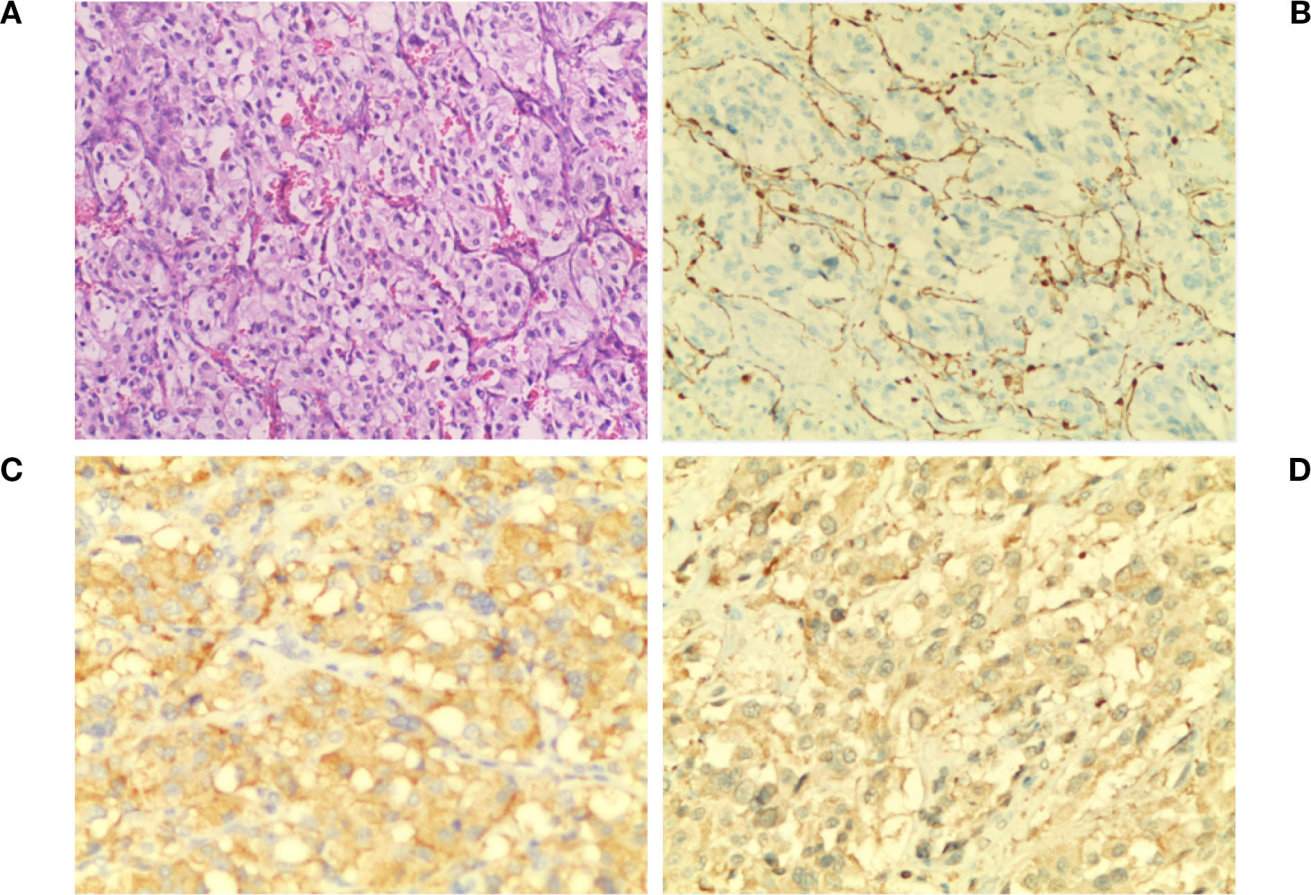
Pathological diagnosis. **(A)**The image shows chief cells arranged in a typical nest-like (zell ballen) glandular pattern. The cells are surrounded by fibrous vascular stroma (red structures in the background) and scattered supporting cells (H&E ×100). **(B)** S-100 staining reveals brown fibrous septa in the tumor stroma with brown cytoplasm and light blue nuclei (×100). **(C)** Syn staining shows evenly distributed, moderately intense brown granules in the cytoplasm and clearly defined fibrous vascular septa in the stroma (×200). **(D)**. CgA staining reveals diffusely distributed, dark brown and brown granules in the cytoplasm with light blue nuclei. The staining is uniform and well-defined (×200).

The patient exhibited symptoms consistent with a neurogenic tumor. Preoperative optimization was conducted, with multidisciplinary consensus for surgical resection. The patient underwent a thoracoscopic-assisted left posterolateral thoracotomy under general anesthesia. Intraoperative exploration revealed a 10 × 7 × 5 cm (**Figure 2A**) ovoid solid mass at the left anterolateral to the descending aorta, exhibiting dense fibrous adhesions to the aortic adventitia and posterior chest wall, with firm consistency and limited mobility (**Figure 2B**). **Figure 3** displays the SBP/DBP, HR, drug infusion rate, and bolus timing at critical perioperative junctures in a timeline. Tumor manipulation triggered a hypertensive crisis (235/132 mmHg, meeting “very high-risk” criteria per ESC Hypertension Guidelines) with concurrent tachycardia (HR 179 bpm), prompting immediate suspicion of paraganglioma. Intraoperative hypertensive crisis (235/132 mmHg) triggered by tumor manipulation prompted an emergent catecholamine assay. A dual-channel regimen (nitroprusside + nitroglycerin + phentolamine) with esmolol was achieved, resulting in hemodynamic stabilization (140-110/90–60 mmHg) within 15 minutes under IBP/CVC guidance. Vascular control was achieved through the utilization of Hem-o-lock/silk ligation. Post-venous ligation hypotension (61/30 mmHg) required the administration of metaraminol and norepinephrine. The en bloc resection was completed via meticulous dissection of aortic adventitial adhesions (**Figures 2A, C**). Post-tumor resection hypotension (36/26 mmHg) necessitated a volume-vasoactive stepwise protocol, involving rapid infusion of crystalloid and colloid solutions (hydroxyethyl starch 500 mL + lactated Ringer’s 500 mL + sodium bicarbonate 100 mL) with concomitant administration of norepinephrine (**Figure 3**). The provision of advanced hemodynamic support post-ICU resulted in the restoration of stabilized hemodynamics within a 48-hour timeframe. The histopathological analysis of the tissue sample confirmed the presence of paraganglioma. The immunohistochemical analysis revealed the presence of Chromogranin A and Synaptophysin, with sustentacular cells S100 positive (**Figure 4**). Intraoperative plasma norepinephrine (NE) levels reached: 3,497.3 pg/mL. On the first postoperative day, NMN levels decreased to 512.0 pg/mL. By one week postoperatively, NMN levels had decreased further, to 43.7 pg/mL. A six-month follow-up period was conducted, during which patients were regularly monitored for blood pressure, heart rate, plasma normetanephrine (NMN) levels in urine, and imaging tests. No abnormalities were detected.

## 3 Discussion

Posterior mediastinal tumors are predominantly neurogenic, while paragangliomas are rare and often misdiagnosed. Characteristic symptoms of functional paragangliomas include episodic hypertension accompanied by headaches, sweating, and palpitations, which are caused by the sudden release of catecholamines (10). Patients exhibiting symptoms of sympathetic hyperactivity (e.g., palpitations, sweating, and hypertension) should undergo routine screening for catecholamine levels. Elevated levels of free norepinephrine and 3-methoxyepinephrine in plasma or 24-hour urine (greater than twice the upper limit of the normal range) are typically diagnostic (11). ^68^Ga-DOTATATE PET/CT can use targeted radioactive tracers to precisely localize paragangliomas and differentiate them from schwannomas (12). Surgical Challenges and Reflections: Surgery for posterior mediastinal paraganglioma requires careful consideration of hemodynamic control and the separation of adhesions in critical areas. A thoracoscopic-assisted minimally invasive thoracotomy approach was employed to ensure precise surgical manipulation while maintaining the capability to manage significant blood loss. Technical reflections: Firstly, Hem-o-lock clips used to occlude the aortic supply branch are prone to dislodgement when blood pressure is elevated. Consequently, silk ligation is recommended to enhance stability. Secondly, preoperative selective embolization of the tumor-supplying arteries may reduce the risk of hypertension crises and bleeding during surgery.

### 3.1 Perioperative management

Acute cardiovascular events are a leading cause of death in PPGL patients (5). Common triggers for catecholamine crises include anesthesia, tumor manipulation, physical activity, and many medications, such as opioids, metoclopramide, and glucagon (10). Therefore, all patients with pheochromocytoma or paraganglioma should undergo thorough preoperative preparation. The first step is to stabilize blood pressure, aiming for a reading below 130/80 mmHg. Anti-hypertensive medication should be started at least 14 days before surgery. If blood pressure is not adequately controlled, surgery should be postponed (2, 13). Major consensus guidelines state that alpha-adrenergic receptor blockers, such as doxazosin or phenoxybenzamine, are the preferred drugs for reducing perioperative complications (1, 2). For patients with PPGL and uncontrolled hypertension despite taking α-adrenergic receptor blockers, calcium channel blockers are the recommended additional drug class (14). Secondly, controlling the heart rate is another important goal of perioperative management. The recommended target heart rate is 60–70 beats per minute when sitting and 70–80 beats per minute when standing. It is important to note that β-adrenergic blockers must never be administered before α-blockers, as this can trigger a hypertensive crisis (11, 15). Patients with heart rates within the target range do not require beta-blocker therapy (13). Pediatric patients require longer treatment times for blood pressure and heart rate to return to normal than adults do. Therefore, treatment should be initiated at least 14 days before surgery compared to adults. In addition to antihypertensive medications, 6–10 g/day of sodium salts and adequate fluid resuscitation are essential, with fluid intake increased to 1.5 times the body weight-corrected level (16). Despite the administration of adequate preoperative treatment, hypertensive crises in patients with PPGL primarily occur during tumor manipulation during surgery. Therefore, continuous invasive blood pressure monitoring via an arterial catheter during surgery is imperative to assist anesthesiologists in evaluating blood pressure fluctuations (17). If a hypertensive crisis occurs during surgery, short-acting antihypertensive drugs must be administered intravenously (16). As paraganglioma was not a preoperative consideration in this case, relevant biochemical screening and α/β-blocker preparation were not performed. Fortunately, a collaborative management model between the thoracic surgery and anesthesiology departments was promptly initiated upon high suspicion during surgery, establishing a hemodynamic management protocol of “real-time monitoring, dynamic adjustment, and precise intervention.” This ultimately resulted in complete resection and safe postoperative recovery. Postoperative hypotension, which is multifactorial in nature and responsive to colloid or crystalloid infusion, may occur due to a combination of factors. These factors include tumor resection, the subsequent downregulation of adrenaline receptors, the acute withdrawal of catecholamines, the long-term effects of preoperative antihypertensive medications, and the short-term effects of intraoperative management (17, 18). Monitoring of blood pressure, heart rate, respiration, urine output and oxygen saturation is required in the intensive care unit (ICU), along with volume resuscitation and vasopressor support.

### 3.2 Follow-up

Research has demonstrated that hereditary PPGL is more prevalent in children and frequently manifests as multifocal, metastatic, or recurrent tumors. Genetic susceptibility to PPGL is generally linked to particular gene mutations. Notable susceptibility genes encompass *VHL, SDHA, SDHB, SDHC, SDHD, RET, NF1, TMEM127, MAX, HRAS*, and others. The occurrence of mutations in these genes has been demonstrated to frequently disrupt the catecholamine synthesis pathway, thereby increasing the risk of tumorigenesis. According to the NCCN guidelines, genetic testing is recommended for adolescents with PPGL who have *SDHB* mutations (19). However, the patient declined to undergo genetic testing due to personal reasons. Given the potential for local recurrence, multifocal disease, or metastatic disease, postoperative care monitoring is imperative for all patients with pheochromocytoma and paraganglioma (PPGL) (20). For all functional PPGLs (paragangliomas with elevated preoperative levels), the measurement of plasma or urine levels of free norepinephrine and 3-methoxytyramine is recommended two to six weeks after surgery (1). Patients considered to be at high risk (i.e., those who are young, have genetic disorders, primarily *SDHB* mutations, have a pheochromocytoma larger than 5 cm, and/or have paragangliomas of any size) should undergo annual imaging screening during the first 5 years, preferably using an MRI to minimize radiation exposure. Subsequent to the initial screening, it is recommended that subsequent screenings be performed at intervals of one to two years (13).

### 3.3 Clinical implications of this case

For adolescent posterior mediastinal masses, even in the absence of persistent hypertension, paroxysmal symptoms such as exercise-induced palpitations warrant prioritizing PPGL in preoperative differential diagnosis and prompt completion of MN/NMN biochemical screening. We advocate a tripartite evaluation combining clinical presentation, anatomical imaging (contrast-enhanced CT/MRI), and biochemical markers (MN/NMN) as the primary screening approach. For high-risk or suspicious cases, functional imaging (^68^Ga-DOTATATE/MIBG) and genetic testing should follow to assess multifocal/metastatic potential and hereditary risk, thereby optimizing surgical approach and perioperative strategies. Given the higher risk of intraoperative hemodynamic fluctuations and bleeding in functional PPGL, thorough preoperative alpha-adrenergic blockade and volume loading are essential. During surgery, the anesthesia team should implement target-directed, precision circulatory management to maintain blood pressure and perfusion stability, thereby enhancing safety during exposure, ligation, and hemostasis of critical great vessels. High-risk cases should undergo perioperative care within a coordinated system involving thoracic surgery, cardiac surgery, anesthesia, and ICU. This case lacked specialized preoperative biochemical/functional imaging assessment for PPGL, highlighting persistent blind spots in identifying atypical/oligosymptomatic adolescent PPGL. Future screening should be prioritized during outpatient visits or early hospitalization.

## 4 Conclusion

This case exemplifies the substantial diagnostic and surgical management challenges posed by a posterior mediastinal paraganglioma. Preoperative multimodal imaging and biochemical assessments were used to determine tumor localization and functional status. The perioperative management strategy centered on alpha-blockade, and the intraoperative regulation of blood pressure effectively mitigated severe fluctuations in blood flow. This case underscores the significance of multidisciplinary collaboration in the management of rare tumors.

## Data Availability

The original contributions presented in the study are included in the article/supplementary material. Further inquiries can be directed to the corresponding author.
